# 2566. Effect of CYP2C19 on Voriconazole Pharmacokinetics in Children with Malignancy or Inborn Errors in Immunity

**DOI:** 10.1093/ofid/ofad500.2183

**Published:** 2023-11-27

**Authors:** Kensuke Shoji, Keiko Hikino, Jumpei Saito, Toshihiro Matsui, Tomoyuki Utano, Akira Takebayashi, Daisuke Tomizawa, Motohiro Kato, Kimikazu Matsumoto, Takashi Ishikawa, Toshinao Kawai, Hidefumi Nakamura, Isao Miyairi, Chikashi Terao, Taisei Mushiroda

**Affiliations:** National Center for Child Health and Development, Setagaya, Tokyo, Japan; RIKEN Center for Integrative Medical Sciences, Yokohama, Kanagawa, Japan; Natoinal Center for Child Health and Development, Setagaya-ku, Tokyo, Japan; National Center for Child Health and Development, Setagaya, Tokyo, Japan; National Center for Child Health and Development, Setagaya, Tokyo, Japan; National Center for Child Health and Development, Setagaya, Tokyo, Japan; National Center for Child Health and Development, Setagaya, Tokyo, Japan; National Center for Child Health and Development, Setagaya, Tokyo, Japan; National Center for Child Health and Development, Setagaya, Tokyo, Japan; National Center for Child Health and Development, Setagaya, Tokyo, Japan; National Center for Child Health and Development, Setagaya, Tokyo, Japan; National Center for Child Health and Development, Setagaya, Tokyo, Japan; Hamamatsu University School of Medicine, Hamamatsu, Shizuoka, Japan; RIKEN Center for Integrative Medical Sciences, Yokohama, Kanagawa, Japan; RIKEN Center for Integrative Medical Sciences, Yokohama, Kanagawa, Japan

## Abstract

**Background:**

Voriconazole pharmacokinetics (PK) are known to be affected by genetic polymorphisms of drug-metabolizing enzymes such as *CYP2C19*; however, such information is limited for the pediatric population.

**Methods:**

This was a single-center population PK study. Information regarding patient background, laboratory test data, voriconazole serum concentration, voriconazole dosing status, and concurrent medications were extracted from electronic medical records. *CYP2C19* genotypes was assessed by whole genome genotyping and defined as follows: *1/*1, *1/*17: normal metabolizer [NM], *1/*2, *1/*3: intermediate metabolizer [IM], and *2/*2, *2/*3, *3/*3: poor metabolizer [PM]. Population PK analysis was performed using Phoenix NLME (v8.3.0). The voriconazole serum concentration profile was described by a two-compartment model with first-order absorption, mixed linear and nonlinear (Michaelis-Menten) elimination, and allometric scaling, as described in previous reports. Covariates were examined using a stepwise method and included to the model when the objective function [-2LL (log-likelihood)] by the maximum likelihood method decreased significantly (Δ-2LL > 6.63, p < 0.01 by likelihood ratio test).

**Results:**

Voriconazole concentration data were available from 60 patients with a median age of 5.3 years. In total, 526 measurements were available for population PK analysis. The phenotypes predicted from *CYP2C19* genotypes were NM in 22 patients, IM in 27 patients, and PM in 11 patients. Underlying diseases included 40 patients with malignancy (66.7%), 18 patients with inborn errors of immunity (30.0%), and 2 patients with other comorbidities. Among the *CYP2C19* phenotypes, PM was predicted to show complete inhibition (the degree of Vmax inhibition (Vmax,inh) = 100%; Vmax= 0). The estimated parameters of Vmax,inh were +0.8 higher in patients with γ-GTP Grade 2 or higher compared with those with Grade 1 or lower. The increase in Vmax,inh was shown to be caused by an increase in C-reactive protein (CRP); the estimated parameters of Vmax,inh were +2.7 higher when CRP was 2.0 mg/dL or higher.

**Conclusion:**

*CYP2C19* genetic polymorphisms (PM), γ-GTP, and CRP affect Vmax,inh of voriconazole in Japanese children with malignancy or inborn errors in immunity

**Disclosures:**

**Kensuke Shoji, MD, PhD**, AstraZeneca K.K.: Honoraria|Gilead Sciences, Inc.: Honoraria|KYORIN Pharmaceutical Co.,Ltd.: Honoraria|Meiji Seika Pharma Co., Ltd.: Honoraria|Nippon Becton Dickinson Company, Ltd.: Honoraria|Novartis Pharma Co., Ltd.: Honoraria|Viatrs, Inc: Honoraria **Motohiro Kato, MD, PhD**, Amgen: Honoraria|Bayer: Honoraria|Chugai Pharm: Honoraria|CSL Behring: Honoraria|Daiichi Sankyo: Grant/Research Support|Konica Minolta REALM: Honoraria|Kyowa KIRIN: Honoraria|Maruho: Honoraria|Nippon Shinyaku: Honoraria|Novartis: Honoraria|Ohara Pharma: Honoraria|Riken Genesis: Honoraria|Sanofi: Honoraria|Sumitomo Pharma: Honoraria|Takeda Pharma: Honoraria **Toshinao Kawai, MD, PhD**, Novartis Pharmaceuticals: Honoraria|Takeda Pharmaceutical Company: Advisor/Consultant|Takeda Pharmaceutical Company: Honoraria **Hidefumi Nakamura, MD, PhD**, Asahi Kasei Corporation: Stocks/Bonds|Bristol Myers Squibb Company: Honoraria|Chugai Pharmaceutical Co., Ltd.: Honoraria|Daiichi Sankyo Company, Ltd.: Advisor/Consultant|Pfizer Global Supply Japan Inc.: Advisor/Consultant|Pfizer R&D Japan G.K.: Advisor/Consultant|Sato Pharmaceutical Co., Ltd.: Advisor/Consultant|Shionogi & Co., Ltd.: Honoraria|Taisho Pharmaceutical Holdings Co., Ltd: Advisor/Consultant **Isao Miyairi, MD, PhD**, Astrazeneca: Honoraria|Mitsubishi-Tanabe: Honoraria|Pfeizer: Honoraria|Sanofi: Advisor/Consultant|Shionogi: Honoraria **Taisei Mushiroda, PhD**, Zenyaku Kogyo Company, Limited: Grant/Research Support

